# Microvascular free flap coverage of complex soft tissue defects after revision total knee arthroplasty: a cross-sectional observation study

**DOI:** 10.2340/17453674.2024.40183

**Published:** 2024-04-17

**Authors:** Nizar HAMROUNI, Jens H HØJVIG, Ulrik K KNUDSEN, Kurt K SKOVGAARD, Lisa T JENSEN, Christian T BONDE, Anders ODGAARD

**Affiliations:** 1 Department of Plastic Surgery and Burns Treatment, Center of Head, Neck and Orthopedics, University Hospital of Copenhagen, Rigshospitalet, Copenhagen; 2 Department of Orthopedic Surgery, Center of Head, Neck and Orthopedics, University Hospital of Copenhagen, Rigshospitalet, Copenhagen; 3 Department of Clinical Medicine, University of Copenhagen, Copenhagen, Denmark

## Abstract

**Background and purpose:**

Soft tissue defects after total knee arthroplasties (TKA) represent a major orthopedic challenge with amputation as a feared outcome. Microvascular free flap coverage (FFC) can increase limb salvage rates, but complications related to the procedure are yet to be explored further. We aimed to review a single-center experience with FFC for soft tissue defects related to revision total knee arthroplasty.

**Methods:**

Through a retrospective chart review from 2006 to 2021, we identified all patients who had FFC of a knee with an existing TKA. Typically, patients underwent 2-stage revision arthroplasty. To identify areas of intervention, we divided the entire regimen into 2 phases divided by the free flap surgery (pre- and post-free flap).

**Results:**

We identified 18 patients with a median age at free flap surgery of 69 years (range 39–85), who were followed for a median of 5.1 years (range 2 months to 10.6 years). The median duration from primary TKA to their final operation was 17.5 months (range 19 days to 7 years). Patients underwent a mean of 7.6 surgical procedures on their knee with 3.6 orthopedic revisions prior to the FFC and 0.6 after. Soft tissue coverage was achieved in all patients and no patients underwent amputation. One-third of patients experienced early complications at recipient site after free flap surgery. There were no donor site complications.

**Conclusion:**

Microvascular FFC of complex soft tissue defects after revision total knee arthroplasty proved achievable in all patients with successful limb salvage in all patients.

Primary total knee arthroplasty (TKA) is associated with complications that require revision and occur in about 6% of cases within 10 years [1]. One of the most frequent causes of revision TKA is periprosthetic joint infection (PJI) [2]. Successful treatment of PJI after TKA involves surgical intervention and antibiotic treatment as either open debridement, antibiotics, and implant retention (DAIR), 1-stage or 2-stage revision [3]. All 3 strategies have a high risk of complications including persistent infection and soft tissue breakdown, necessitating resection arthroplasty, arthrodesis, or amputation [4-6].

Inadequate soft tissue cover may lead to salvage procedures [7,8]. Optimal treatment of such soft tissue defects requires orthopedic and plastic surgical collaboration. The most commonly applied local flap to cover soft tissue defects around the knee is the gastrocnemius muscle flap [9]. However, the gastrocnemius muscle flap is limited in size, and microvascular free flap coverage (FFC) often constitutes the best reconstructive option to achieve sufficient and well-vascularized soft tissue cover [10].

The scarcity of literature on this topic may limit the propagation of FFC. This study therefore has the goal of improving our understanding of the subject, decreasing heterogeneity of approaches, and ensuring awareness of the importance of considering early referral and ultimately reducing the rate of complications leading to arthrodesis or amputation.

The purpose of this cross-sectional case series study is to review our experience with microvascular free flap coverage of soft tissue defects after revision total knee arthroplasty, describing indications and limb salvage outcome.

## Methods

The manuscript is reported according to STROBE’s guidelines.

### Patient selection

A retrospective chart review of the last 15 years (January 2006 to January 2021) was conducted on all patients who had a free flap transfer to cover a soft tissue defect in a knee with an existing KA. We found that all eligible patients had a total knee arthroplasty (TKA). Patients were included if the indication for TKA had been either primary osteoarthritis (OA), secondary OA, or rheumatoid arthritis. Patients who had had a primary TKA due to malignancy or trauma were excluded. 18 patients were included in this study. All patients were treated at our highly specialized tertiary care unit and were mainly operated on by the same surgeons.

We collected retrospective data from clinical records, including age, body mass index, smoking status, alcohol consumption, diabetes mellitus status, and duration of relevant periods. Orthopedic procedural data and complications were included. Orthopedic revision was categorized as major and minor revision. Major revision involves the removal and/or replacement of 1 or both major arthroplasty components (tibial or femoral). Minor revision was defined as the insertion, removal, and/or replacement of any other component or implant including patellar resurfacing [11]. Other procedures, such as debridement, irrigation, or negative pressure wound therapy (NPWT), were also categorized as minor revisions.

Plastic surgical procedural and hospitalization data along with complications were likewise retrieved, as was information regarding previous surgery and infections in the same knee. All periods and durations are reported as medians with ranges, while number of procedures is given as means with a median and range for reference.

### Course of treatment

Patients deemed candidates for free flap surgery had to have completed a 30-day preoperative smoking cessation, unless they were in imminent danger of amputation, as well as complete cessation postoperatively. Preoperative planning of vessel suitability was assessed at the recipient site using angiography (either conventional or CT-based) whereas donor-site vessels were primarily assessed using handheld Doppler ultrasound and in cases supplemented by CT-angiography. The absence of malignant disease and reasonable life expectancy was required.

The surgical course was divided into 2 phases. Phase 1 was defined as the time from primary TKA till the day before free flap surgery. It involved the primary TKA, subsequent revisions, with or without spacer insertion and reimplantation, and the development of a soft tissue defect. Phase 2 was defined as the period from the day of free flap surgery till any final outcome. Final outcomes were either successful prosthesis salvage at the end of follow-up (corresponding to the last observation until January 2021), arthrodesis, amputation, or death. Phase 2 involved the free flap surgery and any concomitant orthopedic procedures (insertion of spacer or reimplantation of prosthesis), and subsequent soft tissue coverage, debridement, spacer revision due to persistent infection, and re-reimplantation.

### Surgical strategy

Patients were initially assessed and deemed candidates for revision knee arthroplasty by a specialist in knee arthroplasty surgery. Criteria for assessment included a combination of clinical, microbiological, biochemical, and radiological, histological, and objective parameters guided by the MSIS criteria of PJI [12]. All patients were infected during phase 1. Patients with a soft tissue defect that could not be simply excised and closed were referred to the plastic surgical department for assessing the possibility of flap coverage. When indicated, revision total knee arthroplasty for infection with free flap soft tissue reconstruction was carried out as an orthoplast joint venture 2-stage revision arthroplasty (Figure).

**Figure:**
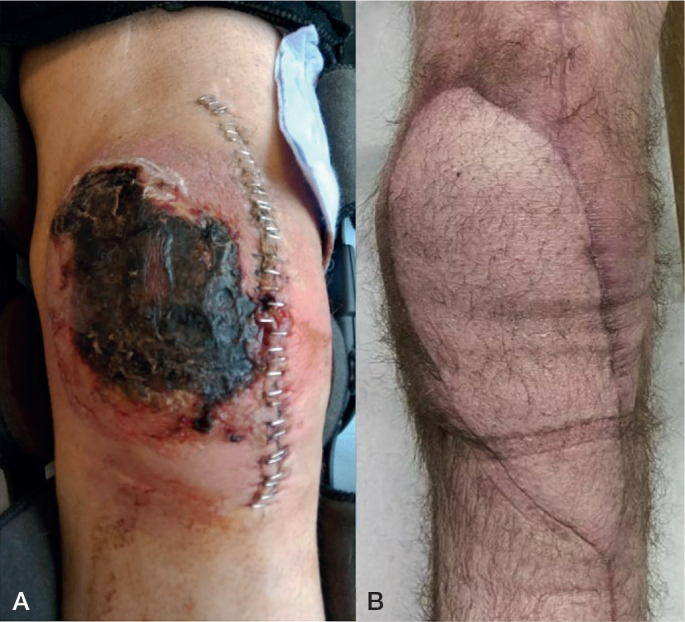
A. Full-skin necrosis with discrete wound dehiscence before orthoplastic joint-venture revision with microvascular free flap coverage and spacer insertion. B. A fully integrated and healed anterolateral thigh (ALT) flap 3 months after the first stage of joint-venture revision at the time of spacer removal and reimplantation arthroplasty.

In the first procedure the orthopedic surgeons carried out removal of the prosthetic components, debridement, irrigation, reaming of medullary canals, and instillation of an antibiotic loaded cement spacer to the knee, and the plastic surgeons harvested a suitable free flap simultaneously. Selection of the appropriate flap (latissimus dorsi or anterolateral thigh flap) for each soft tissue defect was based on an individual assessment of patient and defect characteristics. After the orthopedic revision was carried out, the plastic surgeons prepared the recipient site for microsurgical anastomosis, which was accomplished by anastomosis of the free flap vessels to suitable vessels at the recipient site, often the anterior tibial vessels.

Subject to infection eradication after prolonged susceptibility-guided antibiotics, the orthopedic surgeons elevated the free flap and exchanged the spacer with a permanent arthroplasty during the second procedure. This was often approximately 3 months after the first procedure. In the case of persistent infection, antibiotic spacer exchange was performed.

Antibiotic regimen between the 2 procedures was based on the microbiological profile of the organisms cultured from the first procedure. Antibiotic treatment was continued throughout the 3-month gap and was followed by a 2-week period of discontinuation, to assess the presence of persistent infection prior to reimplantation in the second procedure. Treatment decisions were made by orthopedic surgeons and microbiologists.

Successful soft tissue coverage was defined as complete and intact soft tissue surrounding the knee with concomitant absence of infection as defined by the MSIS criteria.

### Ethics, funding, and disclosures

The study complies with standards for research practice and reporting. The authors have no conflict of interest and received no funding for the study. Complete disclosure of interest forms according to ICMJE are available on the article page, doi: 10.2340/17453674.2024.40183

## Results

18 patients and knees (7 men and 11 women) with a median age at primary TKA of 66 (range 37–81) years were included in this study. The median age at free flap surgery was 69 (range 39–85). Median follow-up from date of free flap surgery was 5.1 years (range 0.2–10.6). All primary knee arthroplasty procedures were total knee arthroplasties.

The median duration from primary TKA to the final operation was 17.5 months (range 0.6–86). The final operation was defined as the last surgical procedure on the same knee or donor site during our follow-up period. The median duration from first revision to final operation was 9.5 months (range 0–85). The total mean number of operations during the entire period was 7.6 procedures including the primary TKA. Patients had a median baseline BMI at insertion of primary TKA of 31 (range 22–40) and 29 (range 18–38) at date of free flap surgery (Table 1).

**Table 1.** General characteristics

General characteristics	Median (range)
Months from TKA to final operation	17.5 (0.6–86)
Months from first revision to final operation	9.5 (0–85)
Follow-up in years from date of free flap surgery	5.1 (0.2–10.6)
Initial age	
Phase 1	66.0 (37–81)
Phase 2	68.5 (39–85)
Difference	2.5
Initial BMI	
Phase 1 (n = 12)	31 (22–40)
Phase 2 (n = 15)	29 (18–38)
Difference	2.1

Follow-up was from date of free flap surgery until date of death or January 12, 2021, corresponding to the date of the last observed observation.

Baseline risk factors prior to FFC included smoking, alcohol consumption, and diabetes mellitus. 8 patients had been continuously smoking until 30 days prior to FFC, while 2 patients were previous smokers. 9 patients had regular intake of alcohol above the nationally established weekly recommendations, while 5 patients described occasional intake within recommendations. Only 2 patients had diabetes mellitus type 2.

### Phase 1

The median duration of Phase 1 was 8.9 months (range 19 days to 7 years). Median time from primary TKA to first revision was 40 days (range 2 days to 5.5 years) and a median period of 71 days (range 0 days to 19 months) between first revision and free flap surgery. During Phase 1, patients underwent a mean of 3.6 revisions and were categorized into 2 groups, first revision within 2 years of primary TKA (n = 15) and after 2 years (n = 3) [2]. Major revision was carried out in 17 patients, while 1 patient solely had minor revision. Of the patients who underwent major revision, 10 patients had an articulated spacer, 2 patients had a non-articulated spacer, while 2 patients had both types implanted at different times. The remaining 3 did not have any spacers.

### Phase 2

The free myocutaneous latissimus dorsi (LD) flap was used in 12 cases. The free fasciocutaneous anterolateral thigh (ALT) flap was chosen in 7 cases.

2 flaps were lost due to arterial thrombosis. 1 patient received a second free flap, while the other received full-thickness skin graft. Successful soft tissue coverage was obtained in all patients.

Patients were hospitalized in the department of plastic surgery (DOPS) for a median of 9.5 days (range 5–32) with drains removed after a median of 6.5 days (range 2–15). 2 patients were discharged from the DOPS, while 16 patients were transferred to an orthopedic department. Median subsequent orthopedic hospitalization was 7 days (range 1–33), accumulating to a median of 18.5 days (range 6– 48) of hospitalization after free flap surgery. Data regarding duration of subsequent orthopedic hospitalization was not accessible in 2 patients (Table 2).

**Table 2:** Phase 2 characteristics

Duration	Median (range)
Months from free flap surgery to final operation	3.5 (0–50)
Hospitalization, days	
Initial LOS (n = 18) ^a^	9.5 (5–32)
Subsequent LOS (n = 16) ^b^	7.0 (1–33)
Total LOS (n = 16) ^c^	18.5 (6–48)
Time to drain removal	6.5 (2–15)
Time to readmission (n = 3)	125 (21–182)
Duration of readmission	8.0 (7–14)

a LOS: Length of stay = duration of hospitalization.

b Subsequent hospitalization includes hospitalization at an orthopedic department after discharge from department of plastic surgery.

c Total LOS = initial LOS + subsequent LOS.

3 patients were readmitted to the DOPS after a median of 125 days (range 21–182). The median duration of readmission was 8 days (range 7–14). Median duration of Phase 2 including the reoperations was 3.5 months (range 0–50) (Table 2).

### Complications and reoperations

Complications within 30 days of FFC that required surgical or antibiotic intervention occurred at the recipient site in 6 patients. Of these, 5 patients had hematomas, 1 patient experienced a total flap loss, and 1 patient a partial flap loss. No patients had complications at the donor site. 1 patient had a systemic complication and developed pseudomembranous colitis. 3 patients were readmitted and reoperated on after 30 days due to development of additional soft tissue defects related to reimplantation of prosthesis.

The patients underwent a mean of 1.3 additional reoperations after the free flap surgery excluding the TKA reimplantation. These were divided into a mean of 0.7 plastic surgical revisions and 0.6 orthopedic revisions (Table 3). The orthopedic revisions in 4 patients were (1) secondary insertion of a revision arthroplasty, (2) soft tissue revision, exchange from articulated to non-articulated spacer as well as re-reimplantation, (3) exchange from articulated to non-articulated spacer and multiple reimplantations due to stem loosening, and (4) soft tissue revision and liner exchange.

**Table 3.** Phase 1 + Phase 2 revisions

Number of revisions per patient	Mean	Median (range)
Phase 1	3.6	3.0 (0–12)
Phase 2	1.3	0.5 (0–8)
Plastic surgical revision ^a^	0.7	0 (0–3)
Orthopedic revision ^b^	0.6	0 (0–7)
Total number of procedures ^c^	7.6	7.5 (2–20)

a Includes the additional free flap.

b Excluding TKA reimplantation.

c Includes primary TKA, revisions Phase 1 + 2, free flap surgery, and TKA reimplantation.

During the follow-up period, 4 patients died. No death was related to the surgical procedure or concomitant hospitalization. Successful prosthesis management with soft tissue reconstruction was achievable in all patients, but 3 patients with successful soft tissue cover died before having their final TKA reimplantation due to, respectively, a malignant disease diagnosed months post-FFC, respiratory distress caused by airway infection months post-FFC, and an unknown cause months post-FFC in a multimorbid patient, all of which postponed their date of reimplantation. 3 patients received additional revision arthroplasty, 1 patient had only minor revision, while 2 underwent regular revision arthroplasty with TKA reimplantation.

## Discussion

We aimed to review a single-center experience with FFC for soft tissue defects related to revision total knee arthroplasty. Successful soft tissue coverage was achieved in all 18 patients, but early plastic surgical complications occurred in one-third of these. Successful final arthroplasty management was obtained in 15 patients. The 3 remaining patients with successful soft tissue cover died before their final arthroplasty reimplantation. No death was related to the surgical procedure or concomitant hospitalization.

Our study identified some characteristics for patients undergoing FFC of soft tissue defects resulting from complicated total knee arthroplasties. Early vascular complications (hematoma and vessel thrombosis) were common. Patients underwent multiple revisions prior to plastic surgical involvement and the possible risk factors, such as BMI and smoking, were present. In this study, soft tissue reconstruction with limb salvage was achieved in all patients.

Few studies have explored the use of free flaps to cover soft tissue defects following infected TKA. Most studies have been smaller series focusing primarily on soft tissue management algorithms as well as limb salvage and flap survival as outcomes [9,13-15].

Lee et al. reviewed 23 microvascular tissue transfers for management of soft tissue defects in infected TKA. During their follow-up period (mean 46 months, n = 22) they found a limb salvage rate of 82% and a median of 2 (range 0–6) additional procedures following coverage and reimplantation [13]. This is comparable to our findings with a limb salvage of 100% and mean revision of 1.3 procedures (median 0, range 0–8) following coverage and excluding final reimplantation. Suda et al. reported limb salvage in 3 out of 5 patients, who had free flaps for soft tissue defects around infected knees [16]. Cetrulo et al. treated 11 soft tissue defects with free LD and rectus abdominis muscle flaps. All flaps survived, with limb salvage in all patients. Prosthesis salvage was obtainable in 82% [10]. Hierner et al. performed 16 LD flaps in 14 patients on prophylactic indication in a young sample (mean age 29 years), where the periprosthetic soft tissue was deemed insufficient prior to prosthetic implantation. The group had a 100% flap survival with 3 patients undergoing arthrodesis due to late infection [17]. Other studies have also reported the use of free flap as a means for successful coverage of periprosthetic soft tissue defects, but reports regarding detailed plastic surgical complications are scarce [15,18–20].

### Risk factors for complications

Previous studies on microsurgical free flap surgery and total knee arthroplasties, individually, have described the relationship of both reconstructive and infectious complications with risk factors such as BMI and smoking.

No studies have established risk factors for complications following FFC of knee arthroplasties, but previous studies on free flaps in breast reconstruction and head and neck surgery have reported that preoperative risk factors such as increased BMI and smoking pose as risks to reconstructive complications [21-23]. In morbidly obese patients (BMI ≥ 40) Watts et al. found that both prepatellar and pretubercal (tibial) soft tissue thickness was associated with infection and early reoperation after regular total knee arthroplasty [24]. Our patient selection and recommendations have been guided by findings in these and previous studies, but further studies on microsurgical reconstructions following TKA are needed to establish a clear association in this group of patients.

### Revision history

Colen et al. presented a study that primarily consisted of local flaps, and found that multiple revisions prior to plastic surgery decreased prosthesis salvage and increased amputation rates [25]. Involving a plastic surgeon early in the course of total knee arthroplasty complications proved beneficial regarding prosthesis salvage and amputation. During Phase 1, our patients underwent a mean of 3.6 orthopedic revisions prior to free flap surgery over a median of 71 days (range 0–19 months). In patients where soft tissue defects are likely to appear, involving a plastic surgeon could therefore be of interest to possibly minimize the number of revisions.

### Complications

Complications were relatively common in our study with one-third experiencing early complications (≤ 30 days) and late complications (> 30 days) happening in 3/18 after FFC. The incidence of early vascular complications related to the flap is higher in our study, while infectious complications are lower and limb salvage higher compared with other studies with similar patients [13,16].

### Flap choice

The myocutaneous latissimus dorsi flap and the fasciocutaneous anterolateral thigh (ALT) flap are both common free tissue transfers used in treating complex soft tissue defects. Both flaps have been used in our study and have different benefits and drawbacks. The LD flap provides sufficient bulk to fill dead space, a long vascular pedicle, covers a large area, and can be raised with a skin paddle [26]. The ALT flap has low donor morbidity and can provide better pliability of the knee. The main drawbacks are the variability in vessels, lack of bulk, and size.

Most other studies also report the use of the LD and ALT flaps and in some cases also the rectus abdominis muscle flaps, but no study has established a superior flap or a convincing algorithm for this patient cohort [18,19].

### Revision approach

The possibility of performing a 1-stage exchange arthroplasty simultaneously with soft tissue reconstruction versus the “gold standard” 2-stage revision is still debatable. If equally effective in eradicating infection, the 1-stage procedure could prove beneficial in terms of economy, time, and burden to patients. Studies have investigated the 1-stage revision approach without free flap surgery and data supports its application in a selected group of patients. Congruity exists in reserving the procedure to the group of patients without major soft tissue deficiencies [27]. A simultaneous 1-stage revision in combination with FFC for treating periprosthetic joint infection with soft tissue loss could be advantageous in terms of reducing the number of revision procedures prior to free flap surgery, but future studies are needed to investigate this approach. Our patients underwent 2-stage revision but, in future patients, performing a 1-stage revision arthroplasty concurrently with free flap coverage may prove beneficial on various parameters and may change the treatment strategy for this patient group. This presupposes equal eradication of infection.

### Strengths and limitations

Although limited by its retrospective nature and heterogeneity of patient regimens as well as no control group, the present study represents one of the most comprehensive reports regarding the use of free flaps to cover soft tissue defects in total knee arthroplasties.

### Conclusion

Our single-center experience with free flap coverage for soft tissue defects related to revision TKA resulted in successful soft tissue coverage in all 18 patients, but one-third of them developed early plastic surgical complications. Nonetheless, it performed effectively as a limb salvage procedure.

In perspective, further studies are needed to clarify uncertainties regarding the revision approach, flap choice, risk factors to complications, and finally to identify factors related to optimization of perioperative care, as in enhanced recovery after surgery protocols [28].

## Notes

NH: Planned the study concept, gathered and analyzed patient data, reviewed the literature, wrote and edited the manuscript including final review. JHH: Planned the study concept, reviewed and edited the manuscript including final review. KKS, LTJ and UKK: Operated on patients, planned the study concept, reviewed the manuscript. AO and CTB: Operated on patients, planned the study concept, reviewed and edited the manuscript including final review.

Handling co-editors: Keijo Mäkelä and Robin Christensen

*Acta* thanks Erik Bülow and Martin Clauss for help with peer review of this manuscript.
